# Usefulness of Chromogenic Media in the Identification of *Candida* spp. Yeasts Compared to Mass Spectrometry

**DOI:** 10.3390/mps8050098

**Published:** 2025-09-01

**Authors:** Agata Bloch, Tomasz Bogiel, Małgorzata Prażyńska, Eugenia Gospodarek-Komkowska

**Affiliations:** 1 Department of Clinical Microbiology, Antoni Jurasz University Hospital No. 1, 85-094 Bydgoszcz, Poland; agatabloch5@gmail.com (A.B.); m.prazynska@cm.umk.pl (M.P.); 2 Department of Propaedeutics of Medicine and Infection Prevention, Nicolaus Copernicus University in Toruń, Ludwik Rydygier Collegium Medicum in Bydgoszcz, 85-067 Bydgoszcz, Poland; 3 Department of Microbiology, Nicolaus Copernicus University in Toruń, Ludwik Rydygier Collegium Medicum in Bydgoszcz, 85-067 Bydgoszcz, Poland; gospodareke@cm.umk.pl

**Keywords:** *Candida* species, chromagar, culture, diagnostic media, MALDI-TOF MS, yeast identification

## Abstract

Yeasts of the *Candida* genus are part of the normal human microbiota but can cause infections (candidiasis) under certain conditions. While *Candida albicans* remains the most common etiological agent, the prevalence of non-albicans *Candida* species—such as *C. glabrata*, *C. tropicalis*, *C. krusei*, *C. parapsilosis*, *C. kefyr*, *C. lusitaniae*, and the emerging multidrug-resistant *C. auris*—has been increasing. Effective treatment of candidiasis requires rapid and accurate identification of the causative species, particularly due to species-specific antifungal agent resistance patterns. The aim of this study was to evaluate the usefulness of five chromogenic media for the differentiation of *Candida* species: BD CHROMagar Candida (Becton Dickinson), CHROM ID Candida (*bio*Mérieux), CHROMAgar Candida Plus (CHROMAgar France, Biomaxima), CHROMAgar Candida Plus (GRASO Biotech), and Brilliance Candida Agar (OXOID). A total of 175 strains from the following species were tested: *C. albicans*, *C. parapsilosis*, *C. dubliniensis*, *C. lusitaniae*, *C. tropicalis*, *C. glabrata*, *C. kefyr*, *C. krusei*, and *C. auris*. Species identification was confirmed by MALDI-TOF mass spectrometry using the MALDI Biotyper system (Bruker). Colony morphology, especially color characteristics, was assessed on each medium. The morphological features of most *Candida* species were consistent with the manufacturer’s descriptions and allowed for presumptive species-level identification. However, some species showed reproducible but previously undescribed morphological traits, including variations in colony shade. Notably, *C. auris* could not be reliably identified using BD, *bio*Mérieux, or OXOID media. In conclusion, while chromogenic media are a helpful preliminary diagnostic tool, subtle differences in colony coloration can complicate interpretation. Diagnostic caution is recommended, and confirmatory methods such as MALDI-TOF remain essential for reliable identification, especially for emerging or less common *Candida* species.

## 1. Introduction

The increasing incidence of systemic fungal infections poses a growing threat to public health worldwide. Global socio-economic development, environmental changes, and pro-ecological practices significantly influence the variability in the occurrence and spread of fungal diseases [1]. Although approximately six million fungal species are estimated to exist globally, only about 1% possess pathogenic potential [2]. These opportunistic pathogens can spread from primary sites of colonization and cause severe secondary infections. The emergence of multidrug-resistant fungal strains further complicates treatment, making the prudent use of antifungal agents a current clinical challenge [1].

Fungi of the *Candida* genus are a natural part of the human microbiota, colonizing mucous membranes of the oral cavity, skin, eyes, reproductive tract, and gastrointestinal system [3,4]. However, under favorable conditions—particularly in immunocompromised individuals or in the presence of biofilm formation—*Candida* spp. representatives can cause serious, sometimes life-threatening infections. Transmission of these pathogens may contribute to complicated disease courses, with various virulence factors playing a role in pathogenesis [2].

*Candida albicans* remains the most frequently isolated species in systemic fungal infections. However, non-albicans *Candida* species, such as *C. parapsilosis*, *C. glabrata*, and *C. tropicalis*, are increasingly recognized as important pathogens, regardless of being less frequent [5].

Rapid and accurate species identification in routine diagnostics is essential for effective treatment and improving patient outcomes. Misidentification or delayed diagnosis can result in treatment failure, particularly in infections caused by species with intrinsic or acquired resistance to antifungal agents such as fluconazole. Prolonged antifungal exposure has also been associated with the emergence of fluconazole-resistant *C. albicans* strains and intrinsically resistant *Candida* species, and further complicating treatment strategies [6].

Although *Candida* species do not produce visible pigments under routine culture conditions, chromogenic media exploit species-specific enzymatic activities to generate distinctive colony colors through cleavage of chromogenic substrates. This mechanism differs from pigment production observed in some filamentous fungi, where colony coloration has direct taxonomic significance.

In this context, chromogenic media represent a practical and cost-effective tool for preliminary identification of *Candida* species, especially in laboratories lacking access to tools of molecular or mass spectrometry-based diagnostics. These media enable presumptive species-level identification within 48 h without requiring advanced instrumentation or specialized training, making them particularly valuable in resource-limited settings [7,8,9,10,11,12].

The urgent need for reliable, accessible diagnostic tools is further underscored by the global emergence of *Candida auris*, an often multidrug-resistant and difficult-to-identify species associated with nosocomial outbreaks and high mortality. Early and accurate recognition of *C. auris* is critical for infection control and targeted antifungal therapy. The newer CHROMagar^TM^ Candida Plus was developed to improve discrimination among non-albicans *Candida* species (NACS) and, critically, to facilitate early recognition of the emerging pathogen *C. auris*. Recent evaluations report high diagnostic accuracy and screening utility: in a set of 303 isolates representing 60 species, CHROMagar Candida Plus enabled accurate differentiation, particularly of *C. auris* [13], and a multicenter study on clinical and environmental surveillance samples found high sensitivity and specificity for *C. auris*, supporting its use for rapid screening and outbreak monitoring [14].

Concordance with conventional methods has also been shown for superficial skin infections, with faster presumptive readouts on chromogenic media [15]. Nevertheless, misidentifications—most commonly involving *C. auris* and *C. glabrata* look-alike phenotypes—have been described, and the conventional CHROMagar Candida exhibits lower sensitivity for several NACS compared with the Plus formulation [16]. These data support the inclusion of chromogenic media as front-line screening tools while underscoring the need for confirmatory methods, such as mass spectrometry or molecular assays, when clinically indicated.

Matrix-assisted laser desorption/ionization time-of-flight mass spectrometry (MALDI-TOF MS) has reformed medical mycology by enabling rapid, species-level identification directly from colonies—excellent for yeasts and increasingly reliable for molds. As reviewed by Lau [7], performance hinges on standardized workflows (e.g., on-plate or full protein extraction) and well-curated spectral libraries. MALDI-TOF MS remains library-dependent: ongoing expansion and updating of reference spectra are essential for resolving cryptic species and improving coverage of filamentous fungi, for which identification can still be variable and benefits from optimized preparation protocols and consensus cutoffs. These developments position MALDI-TOF MS as a first-line identification tool that complements phenotypic methods and, when needed, can be followed by molecular confirmation in clinically critical cases. MALDI-TOF MS was selected for yeast identification in our study due to its high accuracy, speed, and cost-effectiveness in routine microbiological diagnostics. It enables reliable species-level identification of *Candida* spp. within minutes and has been widely validated in clinical microbiology laboratories [7].

The aim of this study was to evaluate the performance of several chromogenic media commercially available worldwide for the identification of clinically relevant *Candida* spp. These media were assessed for their potential application in routine microbiological laboratory-based diagnostics, particularly in settings lacking access to advanced techniques such as MALDI-TOF MS, polymerase chain reaction (PCR), or internal transcribed spacer (ITS) sequencing. The study also tested two novel chromogenic formulations specifically designed for differentiation of *C. auris*, a rarely isolated but clinically significant species.

Most published studies focus on the evaluation of a single chromogenic medium or compare only two. To our knowledge, this is one of the few studies comparing as many as five chromogenic media side-by-side for the identification of clinically significant *Candida* species.

To our knowledge, this is among the few studies that benchmark five chromogenic media head-to-head for clinically significant *Candida* species, including formulations tailored for *C. auris* detection. Most prior reports evaluate a single formulation or compare only two media. Rigorous, side-by-side benchmarking across multiple commercially available options under identical conditions has been lacking. By testing five widely used media in parallel, our study provides actionable, comparative data to inform medium selection for routine workflows and surveillance programs. We further emphasize parameters that matter for routine use—time-to-presumptive identification (24–48 h readouts), ease of interpretation, and potential cost differentials—thereby supporting context-appropriate adoption. The results translate into simple, laboratory-ready guidance on which media best balance sensitivity for non-albicans *Candida* species with the need to flag presumptive *C. auris*.

Our findings help to bridge the gap between high-complexity identification platforms and the day-to-day needs of routine microbiology laboratories. Affordable, ready-to-use chromogenic media remain essential for decentralized laboratories and surge situations, where rapid triage of *Candida* spp. is needed but mass spectrometry or molecular assays are not readily available. Early presumptive recognition of *C. auris* has direct implications for infection prevention and antifungal stewardship, particularly in resource-limited settings.

## 2. Materials and Methods

### 2.1. Strains

The study included 175 clinical strains of *Candida* spp. from the collection of the Department of Clinical Microbiology at Ludwik Rydygier Collegium Medicum in Bydgoszcz, Nicolaus Copernicus University in Toruń, Poland (Table 1). The strains were isolated from clinical specimens obtained from patients at Antoni Jurasz University Hospital No. 1 in Bydgoszcz, Poland, between 2014 and 2021. Detailed data on the origin of the *Candida* spp. isolates are included in the Supplementary Materials (Table S1). The control group consisted of reference strains of *Candida albicans* ATCC 90028 and *Candida auris* ATCC 21092.

### 2.2. Culture Media

Five chromogenic media were evaluated for their ability to differentiate *Candida* species:

- BD CHROMagar Candida (Becton Dickinson, Franklin Lakes, NY, USA).
- CHROM ID Candida (*bio*Mérieux, Marcy-l’Étoile, France).
- CHROMAgar Candida Plus (CHROMagar, La Plaine-Saint-Denis, France and Biomaxima, Poland).
- CHROMAgar Candida Plus (GRASO Biotech, Starogard Gdański, Poland).
- Brilliance Candida Agar (OXOID, Basingstoke, UK).

Each manufacturer provides expected colony morphology descriptions for common *Candida* species. These were used as a reference in this study (Table 2). Representative colony morphology images provided by manufacturers can be found in the Supplementary Materials (Figure S1).

All the applied media were provided by the respective manufacturers free of charge, with the aim of conducting an unbiased comparison to assess diagnostic utility. Batches and expiration dates of CHROMagar products are provided in the Supplementary Materials (Table S2).

### 2.3. Culture and Incubation

Clinical strains were collected between 2014 and 2021, stored at −20 °C, and later subcultured on Sabouraud agar (*bio*Mérieux). All strains were identified to the species level using MALDI-TOF MS with the MALDI Biotyper system (Bruker Daltonics, Bremen, Germany). Identification was performed with the Bruker MALDI Biotyper [MBT Compass 4.1.90 (Bruker, Bremen, Germany)] using the [BDAL/MBT Compass reference library V11.0.0.0 (build 10833)], which includes reference spectra for clinically relevant *Candida* species.

Identification scores ≥ 2.0 were accepted as reliable species-level identification, according to the manufacturer’s criteria. All automated identifications were reviewed by a trained operator against predefined QC criteria, including concordance across replicate spots, inspection of raw spectra and spot quality, and verification of score separation from the next-best match. Scores of 1.7–1.99 were considered genus-level only and prompted repeat testing.

A simplified on-target protein extraction protocol was used. Briefly, a small amount of biomass from a fresh colony was directly applied onto a steel target plate, followed by the addition of 1 µL of 70% formic acid. After air drying at room temperature, 1 µL of matrix solution (α-cyano-4-hydroxycinnamic acid, HCCA, in 50% acetonitrile and 2.5% trifluoroacetic acid) was added. Calibration was performed using the Bruker Bacterial Test Standard (BTS) before each run. For each isolate, two independent on-target preparations (replicate spots) were acquired. Concordant top hits were required for acceptance. Isolates yielding subthreshold scores or non-concordant results were reprocessed (fresh spot or extraction). Representative spectra for *Candida* species are provided in the Supplementary Materials (Figure S2).

MALDI-TOF MS was selected for yeast identification due to its high accuracy, speed, and cost-effectiveness in routine microbiological diagnostics. It enables reliable species-level identification of *Candida* spp. within minutes and has been widely validated in clinical microbiology laboratories [7].

Each confirmed strain was simultaneously inoculated onto all five chromogenic media and incubated under identical conditions (37 °C, up to 72 h). Colony morphology was evaluated at 24, 48, and 72 h. Photographs were taken against both white and dark backgrounds to enhance the visualization of color differences. The most representative images were selected to illustrate distinctive colony characteristics for each medium and species (Supplementary Materials, Figures S3–S7).

## 3. Results

All *Candida* spp. strains were incubated under identical conditions for up to 72 h. Colony morphology and pigmentation were assessed after 24, 48, and 72 h. After 24 h, most colonies exhibited only faint or incomplete coloration. At 48 h, the colony colors were more distinct and typically matched the expected appearance according to the manufacturer’s specifications. Although the primary evaluation and identification were based on the 48 h read-out, incubation was continued to 72 h in order to assess whether any additional phenotypic changes (e.g., pigment intensification, color shift, or delayed differentiation) would occur. This extended incubation allowed for the detection of any late-developing changes that might influence interpretation, although in most cases, no significant alterations were observed beyond 48 h.

Each chromogenic medium was evaluated individually by two investigators, obtaining the same results observations. Observations included colony color, morphology, and any diagnostic challenges. Detailed data are presented in Table 3.

### 3.1. BD CHROMagar Candida (Becton Dickinson)

This medium confirmed the expected characteristics for *C. albicans* (100.0%), *C. tropicalis* (83.3%), and *C. krusei* (100.0%). As reported by the manufacturer, other *Candida* species formed cream-colored to light pink or violet colonies. *Candida dubliniensis* (*n* = 13, 100.0%) displayed morphology identical to *C. albicans*, which is consistent with the noted difficulty in differentiating these closely related species based solely on colonies morphology features.

### 3.2. CHROM ID Candida (bioMérieux)

This medium successfully distinguished *C. albicans* (or *C. dubliniensis*) from other *Candida* species, but it was not capable of differentiating between *C. albicans* and *C. dubliniensis*. Both produced light to medium blue colonies (*n* = 33, 100.0%). Subtle differences were noted, with *C. dubliniensis* appearing slightly lighter in shade, (*n* = 13, 100.0%) a distinction also mentioned in the manufacturer’s documentation. The expected pink coloration for *C. kefyr* (100.0%), *C. lusitaniae* (100.0%), and *C. tropicalis* (88.9%) was observed. *C. auris* strains were pink-white (*n* = 2, 100.0%), and *C. glabrata*, *C. krusei*, and *C. parapsilosis* (*n* = 77, 100.0%) were consistently white; however, these species’ growth characteristics were not specified by the manufacturer.

### 3.3. CHROMAgar Candida Plus (CHROMAgar France)

This medium was effective in differentiating *Candida auris*, which formed light blue colonies with a distinct blue halo, visible especially on the reverse side of the plate. This feature was consistently observed in both clinical isolates (*n* = 2, 100.0%) and the reference strains. As with other media, *C. albicans* and *C. dubliniensis* were indistinguishable. Most species matched the morphological expectations stated by the manufacturer. One isolate of *C. glabrata* showed slightly darker coloration. *C. tropicalis* representatives displayed characteristic bluish-metallic colonies with pink halos, as described in the product documentation. A noteworthy observation was the potential for misidentifying *C. parapsilosis* as *C. auris* due to a similar halo effect in massive growth. However, colony color and distribution helped to differentiate between these species. *C. auris* had consistent halos around individual colonies, whereas *C. parapsilosis* displayed halos only in densely populated areas.

### 3.4. CHROMAgar Candida Plus (GRASO Biotech)

This medium, produced under license from CHROMAgar France, showed nearly identical colony morphology to the product of CHROMAgar France manufacturer. Identification of *C. auris* was possible using the same criteria, including a halo presence and colony color. Morphological features of other species were also consistent, confirming the reliability of this medium in *Candida* species identification.

### 3.5. Brilliance Candida Agar (OXOID)

All the tested species grew on this medium, but differentiation was limited. Most colonies were beige or brown, except for species expressing alkaline phosphatase (*C. albicans*, *C. dubliniensis*, *C. tropicalis*), strains of which produced green or dark blue colonies (Table 3). However, the overall low specificity of this medium hinders accurate identification based solely on morphology.

## 4. Discussion

The increasing incidence of candidemia and invasive fungal infections, especially in immunocompromised patients, highlights the urgent need for rapid and cost-effective tools in clinical microbiology diagnostics [1,8,9]. Chromogenic media serve as valuable first-line tools for presumptive identification of *Candida* species, particularly in resource-limited settings where molecular methods-based diagnostics or MALDI-TOF MS may not be simply available. In the present study MALDI-TOF MS was selected as the reference method for yeast identification due to its high accuracy, speed, and suitability for routine diagnostic use. It enables rapid and reliable species-level identification of *Candida* spp., including uncommon or closely related species. However, despite its advantages, this technique requires a highly specialized and expensive instrument, which may not be readily available in all clinical or microbiological laboratories. As a result, access to MALDI-TOF MS remains limited in some settings, particularly in low-resource environments. Therefore, evaluating alternative methods, such as chromogenic media, remains relevant and valuable in such contexts [10,11,12].

Odds et al. [11] confirmed that CHROMagar Candida (France) afforded the correct presumptive recognition of *C. albicans*, *C. tropicalis*, *C*, *krusei*, and *Trichosporon* spp. Nadeem et al. [12] revealed that CHROMagar Candida (France) can accurately differentiate between *C. albicans*, *C. tropicalis*, and *C. krusei*. The specificity and sensitivity of CHROMagar Candida (France) for *C. albicans* was calculated as 99%, for *C. tropicalis* it was calculated as 98%, and for *C. krusei* it was 100%.

Taverna et al. [13] evaluated the performance of CHROMagar^TM^ Candida Plus for the identification of 303 yeast isolates representing 60 species. The medium demonstrated high diagnostic accuracy, especially for identifying *Candida auris*. However, some isolates were misidentified as *C. auris* or *C. glabrata*, highlighting the limitations of phenotypic methods. Mulet Bayona et al. [14] conducted a multicenter study to assess CHROMagar^TM^ Candida Plus for detecting *C. auris* and other *Candida* species from environmental and clinical surveillance samples. The medium showed high sensitivity and specificity, making it suitable for outbreak monitoring and rapid screening. Rathore et al. [15] compared CHROMagar^TM^ Candida with conventional methods for identifying *Candida* species from superficial skin infections. The study found high agreement between both methods, with CHROMagar offering faster presumptive identification. A study from 2020 [16] compared the diagnostic performance of the new CHROMagar^TM^ Candida Plus with CHROMagar^TM^ Candida. While CHROMagar^TM^ Candida showed good sensitivity overall, it had limitations in identifying species less common than *C. albicans*, such as *C. krusei*, *C. glabrata*, *C. parapsilosis*, *C. orthopsilosis*, *C. lusitaniae*, and *C. auris*.

Our findings demonstrate that CHROMAgar Candida Plus media (France and GRASO Biotech) provided the most reliable morphology-based differentiation, especially for *C. auris*, which exhibited consistent colony features. This is of clinical significance, given the public health concern surrounding this emerging multidrug-resistant species [17,18,19,20,21,22], since the pathogenicity and transmission potential of *C. auris* have raised significant concerns in healthcare settings [19].

A noteworthy observation was the potential for misidentifying *C. parapsilosis* as *C. auris* due to a similar halo effect in crowded cultures, although the subtle differences in colony color and morphology were useful for distinguishing the species when carefully interpreted.

Despite their utility, chromogenic media present notable limitations. Differentiation between *C. albicans* and *C. dubliniensis* remains problematic on all of the tested media due to similar enzymatic profiles [23]. Moreover, species such as *C. tropicalis* showed morphological variability (Table 3) that may affect identification in mixed cultures. This feature might be due to the physiology of *C. tropicalis* and its ability to undergo yeast–hyphae switching [24]. This highlights the need for confirmatory methods in cases of ambiguous results, especially considering the risk of misidentification.

A limitation of this study is the use of laboratory-cultured isolates rather than direct patient specimens, which may differ in colony presentation due to the sample matrix or other microbial presence and their competition.

An important benefit of chromogenic media is their ability to facilitate the early recognition of mixed yeast cultures. Unlike traditional Sabouraud agar, which typically yields homogeneous-appearing colonies, chromogenic media allow differentiation based on colony color and morphology. This advantage significantly improves the detection of polyfungal infections during initial culture evaluation and supports more accurate and timely diagnostic interpretation, particularly in high-throughput clinical settings.

Furthermore, chromogenic media provide an inexpensive and immediate diagnostic option for settings with limited access to molecular methods, improving early treatment decision-making and contributing to antimicrobial stewardship initiatives.

Further evaluation on primary clinical samples, including blood culture-derived yeasts, would be beneficial. Fungal dissemination mechanisms in clinical settings also deserve further exploration, especially in the context of nosocomial outbreaks [2]. Future work could explore the integration of chromogenic media with image analysis for color and morphology classification. Additionally, standardizing colony color interpretation through digital tools could reduce subjectivity and inter-laboratory variability.

In conclusion, chromogenic media remain an accessible and informative component of diagnostic workflows, but their use should be supported by confirmatory methods, especially for less typical, less common, or antifungal-resistant species.

## 5. Conclusions

Chromogenic media represent a valuable, cost-effective, and user-friendly tool for the preliminary differentiation of *Candida* species and early detection of mixed yeast cultures in clinical microbiology laboratories. Among the tested media, CHROMAgar Candida Plus (CHROMAgar France and GRASO Biotech) demonstrated the highest diagnostic accuracy, notably excelling in the identification of *C. auris*, an emerging multidrug-resistant pathogen of significant clinical concern. The distinctive colony morphology and characteristic halo formation further enhance their utility for rapid screening and presumptive diagnosis.

Nevertheless, the inability of chromogenic media to reliably distinguish *C. albicans* from *C. dubliniensis* underscores their inherent limitations, particularly in cases requiring precise species-level identification. For definitive diagnosis, especially in mixed infections or when less common *Candida* species are involved, complementary methods such as MALDI-TOF MS or molecular techniques remain indispensable.

In conclusion, chromogenic media should be regarded as a supportive first-line diagnostic tool, facilitating early recognition and timely initiation of targeted antifungal therapy, while serving as a practical option for laboratories lacking access to advanced identification technologies. Their integration into routine workflows can significantly improve clinical management of candidiasis and contribute to better patient outcomes.

## Figures and Tables

**Table 1:** Number of isolates by species used in the study.

Species	*Candida albicans*	*Candida auris*	*Candida dubliniensis*	*Candida glabrata*	*Candida kefyr*	*Candida krusei*	*Candida lusitaniae*	*Candida parapsilosis*	*Candida tropicalis*
Number of strains	20	2	13	13	13	7	31	57	19
Total	175								

**Table 2:** Expected colony morphology for selected *Candida* species according to manufacturer data (after 48 h incubation at 37 °C).

	Culture Media				
Species	BD CHROMagar *Candida* (Becton Dickinson)	Agar CHROM ID Candida (*bio*Mérieux)	CHROMAgar Candida Plus (CHROMAgar France)	CHROMAgar Candida Plus (GRASO Biotech)	Brilliance Candida Agar (OXOID)
C. *albicans*	light to medium green	light to dark blue	green-blue	green-blue colonies	green
C. *auris*	no data	no data	light blue with blue halo blue from the back side	light blue colonies with blue halo, blue from the back side	no data
C. *dubliniensis*	green, also green for *Candida albicans*	brighter than colonies of *Candida albicans*	no data	no data	green
C. *glabrata*	no data	no data	mauve	purple-pink	beige/yellow/brown
C. *kefyr*	no data	pink	no data	no data	beige/yellow/brown
C. *krusei*	light rose to pink, flat colonies with a whitish border	no data	pink and fuzzy	pink with irregular edges	dry, irregular pink-brown
C. *lusitaniae*	no data	pink	no data	no data	beige/yellow/brown
C. *parapsilosis*	no data	no data	no data	no data	beige/yellow/brown
C. *tropicalis*	blue-gray to blue-greenish or metallic blue colonies with or without violet halos in the surrounding medium	pink	metallic blue with pink halo	metallic, blue with pink halo	dark blue

**Table 3:** *Candida* spp. colony color and morphology observed on selected chromogenic media after 48 h of incubation.

	Culture Media				
Species	BD CHROMagar *Candida* (Becton Dickinson)	Agar CHROM ID Candida (*bio*Mérieux)	CHROMAgar Candida Plus (CHROMAgar France)	CHROMAgar Candida Plus (GRASO Biotech)	Brilliance Candida Agar (OXOID)
C. *albicans*	blue-green (100.0%)	blue (100.0%)	green-blue, aquamarine (100.0%)	green-blue (100.0%)	green, flat, small (100.0%)
C. *auris*	white-cream (100.0%)	pink-white (100.0%)	light blue with blue halo; blue from the back side (100.0%)	light blue with blue halo; blue from the back side (100.0%)	beige, flat (100.0%)
C. *dubliniensis*	blue-green (100.0%)	light blue (100.0%)	green-blue, aquamarine (100.0%)	green-blue (100.0%)	green, flat, small (100.0%)
C. *glabrata*	violet (100.0%)	white, fuzzy (100.0%)	mauve (91.7%)	mauve (91.7%)	beige (91.7%)
			violet-blue (8.3%)	violet-blue (8.3%)	blue-violet (8.3%)
C. *kefyr*	dark beige (100.0%)	pink (100.0%)	mauve (100.0%)	mauve (100.0%)	cream (100.0%)
C. *krusei*	violet-pink (100.0%)	white, fuzzy (100.0%)	violet-pink, fuzzy (100.0%)	violet-pink, fuzzy (100.0%)	beige or pink-brown (100.0%)
C. *lusitaniae*	dark beige (100.0%)	pink (100.0%)	violet (100.0%)	violet (100.0%)	beige (73.4%)
					dark beige (13.3%)
					violet (13.3%)
C. *parapsilosis*	cream-pink (100.0%)	white, small (100.0%)	blue-violet with blue halo (57.4%)	cream-violet with blue halo (90.7%)	beige, flat, small (100.0%)
			blue-violet without blue halo (33.3%)	cream-violet without blue halo (5.6%)	
			blue-violet with violet halo (9.3%)	cream (3.7%)	
C. *tropicalis*	blue-grey (83.3%)	pink (88.9%)	metallic dark blue with pink halo (100.0%)	metallic, blue with pink halo (100.0%)	dark blue or aquamarine, flat, small, shiny (100.0%)
	white-grey (16.7%)	white-blue, small (11.1%)			

## Data Availability

The data presented in this study are available upon request from the corresponding author.

## Supplementary Materials

The following supporting information can be downloaded at https://www.mdpi.com/article/10.3390/mps8050098/s1, Table S1: The origin of the applied *Candida* spp. strains (*n* = 175); Table S2: Batches and expiration dates of CHROMagar products; Figure S1: Representative MALDI-TOF MS spectra for *Candida* species; Figure S2: Representative colony morphology images provided by manufacturers; Figure S3: Photographs of selected cultures on BD CHROMagar Candida by Becton Dickinson: (A) *Candia albicans*, (B) *Candida dubliniensis*, (C) *Candia tropicalis*, (D) *Candida krusei*; Figure S4: Photographs of chosen cultures on Agar CHROM ID Candida by *bio*Mérieux: (A) *Candida albicans*, (B) *Candida dubliniensis*, (C) *Candida tropicalis*, (D1) *Candida tropicalis*, (D2) *Candida lusitaniae*, and (D3) *Candida kefyr*; Figure S5: Photographs of chosen cultures on CHROMAgar Candida Plus by CHROMAgar France: (A) *Candida albicans*, (B) *Candida krusei*, (C) *Candida krusei*, (D) *Candida tropicalis*; Figure S6: Photographs of selected cultures on CHROMAgar Candida Plus by GRASO Biotech: (A,B) *Candida auris*, (C,D) *Candida parapsilosis*; Figure S7: Photographs of some cultures on Brilliance Candida Agar by OXOID: (A) *Candida parapsilosis*, (B) *Candida kefyr*, (C) *Candida lusitaniae*, (D) *Candida krusei*.

## Abbreviations

The following abbreviations are used in this manuscript:

MALDI-TOF	Matrix-Assisted Laser Desorption Ionization–Time-of-Flight Mass Spectrometry
ATCC	American Type Culture Collection

## References

[1] Bongomin F., Gago S., Oladele R.O., Denning D.W. (2017). Global and Multi-National Prevalence of Fungal Diseases—Estimate Precision. J. Fungi.

[2] Strickland A.B., Shi M. (2021). Mechanisms of Fungal Dissemination. Cell. Mol. Life Sci. CMLS.

[3] Ghannoum M.A., Jurevic R.J., Mukherjee P.K., Cui F., Sikaroodi M., Naqvi A., Gillevet P.M. (2010). Characterization of the Oral Fungal Microbiome (Mycobiome) in Healthy Individuals. PLoS Pathog..

[4] Drell T., Lillsaar T., Tummeleht L., Simm J., Aaspõllu A., Väin E., Saarma I., Salumets A., Donders G.G.G., Metsis M. (2013). Characterization of the Vaginal Micro- and Mycobiome in Asymptomatic Reproductive-Age Estonian Women. PLoS ONE.

[5] Pfaller M.A., Diekema D.J. (2007). Epidemiology of Invasive Candidiasis: A Persistent Public Health Problem. Clin. Microbiol. Rev..

[6] Templeton S.P., Rivera A., Hube B., Jacobsen I.D. (2018). Editorial: Immunity to Human Fungal Pathogens: Mechanisms of Host Recognition, Protection, Pathology, and Fungal Interference. Front. Immunol..

[7] Lau A.F. (2021). MALDI-TOF for Fungal Identification. Clin. Lab. Med..

[8] Omrani A.S., Koleri J., Ben Abid F., Daghfel J., Odaippurath T., Peediyakkal M.Z., Baiou A., Sarsak E., Elayana M., Kaleeckal A. (2021). Clinical Characteristics and Risk Factors for COVID-19-Associated Candidemia. Med. Mycol..

[9] McCarty T.P., White C.M., Pappas P.G. (2021). Candidemia and Invasive Candidiasis. Infect. Dis. Clin. N. Am..

[10] Perry J.D., Freydière A.M. (2007). The Application of Chromogenic Media in Clinical Microbiology. J. Appl. Microbiol..

[11] Odds F.C., Bernaerts R. (1994). CHROMagar Candida, a New Differential Isolation Medium for Presumptive Identification of Clinically Important Candida Species. J. Clin. Microbiol..

[12] Nadeem S.G., Hakim S.T., Kazmi S.U. (2010). Use of CHROMagar Candida for the Presumptive Identification of Candida Species Directly from Clinical Specimens in Resource-Limited Settings. Libyan J. Med..

[13] Taverna C.G., Vivot M.E., Arias B.A., Irazu L., Canteros C.E. (2023). Evaluation of the CHROMagar Candida Plus Medium for Presumptive Identification of Yeasts and MALDI-TOF MS Identification. Mycoses.

[14] Mulet Bayona J.V., Salvador García C., Tormo Palop N., Valentín Martín A., González Padrón C., Colomina Rodríguez J., Pemán J., Gimeno Cardona C. (2022). Novel Chromogenic Medium CHROMagarTM Candida Plus for Detection of *Candida auris* and Other Candida Species from Surveillance and Environmental Samples: A Multicenter Study. J. Fungi.

[15] Rathore N., Lalwani J., Singh P., Meena M. (2024). Speciation of Candida Species Isolated from Cutaneous Skin Lesions Using CHROMagar and Conventional Methods. Asian J. Med. Sci..

[16] Mulet Bayona J.V., Salvador García C., Tormo Palop N., Gimeno Cardona C. (2020). Evaluation of a Novel Chromogenic Medium for *Candida* Spp. Identification and Comparison with CHROMagar^TM^ Candida for the Detection of *Candida auris* in Surveillance Samples. Diagn. Microbiol. Infect. Dis..

[17] Plachouras D., Lötsch F., Kohlenberg A., Monnet D.L. (2020). *Candida auris* survey collaborative group *Candida auris*: Epidemiological Situation, Laboratory Capacity and Preparedness in the European Union and European Economic Area*, January 2018 to May 2019. Euro Surveill..

[18] Du H., Bing J., Hu T., Ennis C.L., Nobile C.J., Huang G. (2020). *Candida auris*: Epidemiology, Biology, Antifungal Resistance, and Virulence. PLoS Pathog..

[19] Cristina M.L., Spagnolo A.M., Sartini M., Carbone A., Oliva M., Schinca E., Boni S., Pontali E. (2023). An Overview on *Candida auris* in Healthcare Settings. J. Fungi.

[20] Schelenz S., Hagen F., Rhodes J.L., Abdolrasouli A., Chowdhary A., Hall A., Ryan L., Shackleton J., Trimlett R., Meis J.F. (2016). First Hospital Outbreak of the Globally Emerging *Candida auris* in a European Hospital. Antimicrob. Resist. Infect. Control.

[21] Jeffery-Smith A., Taori S.K., Schelenz S., Jeffery K., Johnson E.M., Borman A., Manuel R., Brown C.S., *Candida auris* Incident Management Team (2018). Candida auris: A Review of the Literature. Clin. Microbiol. Rev..

[22] Bao J.R., Master R.N., Azad K.N., Schwab D.A., Clark R.B., Jones R.S., Moore E.C., Shier K.L. (2018). Rapid, Accurate Identification of *Candida auris* by Using a Novel Matrix-Assisted Laser Desorption Ionization-Time of Flight Mass Spectrometry (MALDI-TOF MS) Database (Library). J. Clin. Microbiol..

[23] Sullivan D., Coleman D. (1998). Candida Dubliniensis: Characteristics and Identification. J. Clin. Microbiol..

[24] Kadosh D., Mundodi V. (2020). A Re-Evaluation of the Relationship between Morphology and Pathogenicity in Candida Species. J. Fungi.

