# Supplementary material for: Usefulness of Chromogenic Media in the Identification of Candida spp. Yeasts Compared to Mass Spectrometry
Source: Methods Protoc. 2025 Sep 1;8(5):98. doi: 10.3390/mps8050098 (PMC12452300; doi:10.3390/mps8050098)

methods and protocols

MDPI

Supplementary Materials

Usefulness of chromogenic media in the identification of *Candida* spp. yeasts compared to mass spectrometry

Agata Bloch [1], Tomasz Bogiel [2,*], Małgorzata Prażyńska [3] and Eugenia Gospodarek-Komkowska [3]

[1] Department of Clinical Microbiology, Antoni Jurasz University Hospital No. 1, 85-094 Bydgoszcz, Poland
[2] Department of Propaedeutics of Medicine and Infection Prevention, Nicolaus Copernicus University in Toruń, Ludwik Rydygier Collegium Medicum in Bydgoszcz, 85-067 Bydgoszcz, Poland
[3] Department of Microbiology, Nicolaus Copernicus University in Toruń, Ludwik Rydygier Collegium Medicum in Bydgoszcz, 85-067 Bydgoszcz, Poland
* Correspondence: t.bogiel@cm.umk.pl

Table S1. The origin of the applied *Candida* spp. strains (n = 175).

Serial No.	Collection No.	Species	Specimen
1.	7272	*Candida albicans*	pus sample
2.	7260		blood sample
3.	7405		urine sample
4.	27775		peritoneal fluid
5.	26786		peritoneal fluid
6.	27046		peritoneal fluid
7.	27968		peritoneal fluid
8.	27738		peritoneal fluid
9.	27451		sinus drainage
10.	27334		blood sample
11.	26022		peritoneal fluid
12.	26567		peritoneal fluid
13.	26710		blood sample
14.	37821		blood sample
15.	38024		prosthesis
16.	38075		pus sample
17.	38425		blood sample
18.	37711		blood sample
19.	ATCC 90028		reference strain
20.	DSM 1386		reference strain
21.	5616 / 2019	*Candida auris*	blood sample
22.	DSM 21092		reference strain
23.	5863	*Candida dublinienis*	blood sample
24.	5920		wound swab
25.	7189		blood sample

26.	7252		drainage fluid
27.	7280		tongue swab
28.	6835		blood sample
29.	6624		pericardial fluid
30.	6715		bronchoalveolar lavage
31.	6784		tip of the catheter
32.	6716		stool sample
33.	6886		peritoneal fluid
34.	7065		tissue sample
35.	6908		blood sample
36.	7227	*Candida glabrata*	bronchoalveolar lavage
37.	7228		blood sample
38.	7279		blood sample
39.	7271		prosthesis
40.	7261		blood sample
41.	7220		blood sample
42.	7223		tip of the catheter
43.	7274		blood sample
44.	7263		bronchoalveolar lavage
45.	7256		bronchoalveolar lavage
46.	7241		peritoneal fluid
47.	7235		tissue sample
48.	5621		blood sample
49.	5191	*Candida kefyr*	urine sample
50.	6824		peritoneal fluid
51.	6882		blood sample
52.	7179		bile sample
53.	5218		bronchoalveolar lavage
54.	5187		urine sample
55.	5450		eye swab
56.	5684		urine sample
57.	5820		wound swab
58.	6720		peritoneal fluid
59.	5632		urine sample
60.	5530		stool sample
61.	5506		drainage fluid
62.	7234	*Candida krusei*	surgical site infection swab
63.	7210		bronchoalveolar lavage
64.	7151		peritoneal fluid
65.	7147		surgical site infection swab
66.	7131		wound swab
67.	7250		bronchoalveolar lavage

68.	ATCC 6258		reference strain
69.	5274	Candida lusitaniae	organ preservation solution
70.	5277		peritoneal fluid
71.	5614		rectal swab
72.	5655		peritoneal fluid
73.	5668		peritoneal fluid
74.	5841		urine sample
75.	6558		urine sample
76.	6869		surgical site infection swab
77.	6594		peritoneal fluid
78.	5226		urine sample
79.	4997		blood sample
80.	6866		peritoneal fluid
81.	6864		peritoneal fluid
82.	6565		rectal swab
83.	6591		peritoneal fluid
84.	5039	*Candida lusitaniae*	wound swab
85.	5055		blood sample
86.	5247		blood sample
87.	4660		bronchoalveolar lavage
88.	4726		peritoneal fluid
89.	5627		blood sample
90.	6393		urine sample
91.	6443		peritoneal fluid
92.	5857		urine sample
93.	6955		peritoneal fluid
94.	6746		urine sample
95.	5859		urine sample
96.	6274		urine sample
97.	5310		blood sample
98.	6245		bronchoalveolar lavage
99.	7188		blood sample
100.	4927		blood sample
101.	4928		blood sample
102.	4986		ear swab
103.	5000		blood sample
104.	5027	*Candida parapsilosis*	wound swab
105.	5031		urine sample
106.	5081		wound swab
107.	5146		wound swab
108.	5167		wound swab
109.	5169		blood sample

110.	5170	blood sample
111.	5179	blood sample
112.	5189	ear swab
113.	5244	tip of the catheter
114.	5301	ear swab
115.	5675	pound swab
116.	5706	blood sample
117.	5739	vaginal swab
118.	5768	chronic wound swab
119.	5770	vaginal swab
120.	5780	urine sample
121.	5792	blood sample
122.	5803	urine sample
123.	5833	tip of the catheter
124.	5837	blood sample
125.	5940	blood sample
126.	5941	blood sample
127.	5942	blood sample
128.	6105	blood sample
129.	6130	urine sample
130.	6131	wound swab
131.	6165	pus sample
132.	6166	wound swab
133.	6361	blood sample
134.	6462	peritoneal fluid
135.	6498	wound swab
136.	6640	peritoneal fluid
137.	6707	urine sample
138.	6737	rectal swab
139.	6744	peritoneal fluid
140.	6751	blood sample
141.	6769	tip of the catheter
142.	6791	blood sample
143.	6792	blood sample
144.	6805	blood sample
145.	6806	blood sample
146.	6809	blood sample
147.	6811	blood sample
148.	6813	blood sample
149.	6822	blood sample
150.	6844	blood sample
151.	6861	blood sample

152.	6879		wound swab
153.	6901		wound swab
154.	6915		blood sample
155.	6924		blood sample
156.	6927		blood sample
157.	6451	*Candida tropicalis*	wound swab
158.	6548		bronchoalveolar lavage
159.	6593		wound swab
160.	6601		urine sample
161.	6606		rectal swab
162.	6713		prosthesis
163.	6725		blood sample
164.	6735		urine sample
165.	6763		blood sample
166.	6784		tip of the catheter
167.	6817		peritoneal fluid
168.	6860		blood sample
169.	6897		blood sample
170.	6941		peritoneal fluid
171.	6994		bronchoalveolar lavage
172.	7029		blood sample
173.	7120		blood sample
174.	7194		sinus drainage
175.	7005		tissue sample

Table S2. Batches and expiration dates of Chromagar products.

**Medium:**	**BD CHROMagar *Candida*** (**Becton Dickinson**)	**Agar CHROM ID Candida** (***bio*Mérieux**)	**CHROMAgar Candida Plus** (**CHROMAgar France**)	**CHROMAgar Candida Plus** (**Graso**)	**Brilliance Candida Agar** (**Oxoid**)
**LOT:**	1019238	1008462170	10255270101RB	20210202	3223412
**Expiration date:**	2021-04-23	2021-03-30	2021-04-07	2021-05-03	2021-03-25

Figure S1. Representative colony morphology images provided by manufacturers.

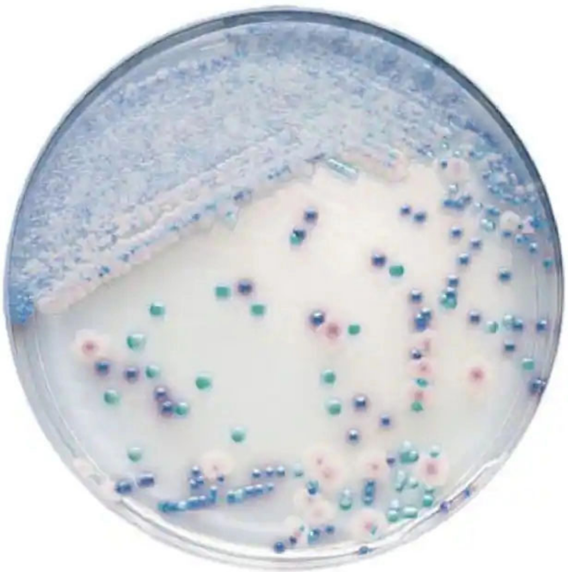

A. BD CHROMagar Candida (Becton Dickinson)

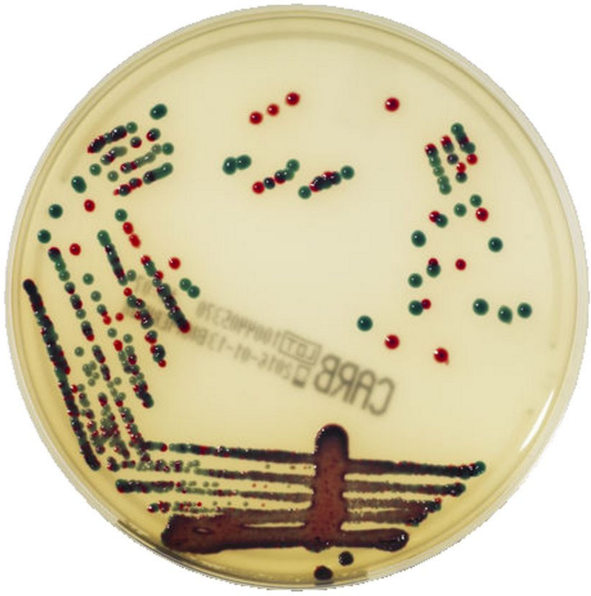

B. Agar CHROM ID Candida (*bio*Mérieux)

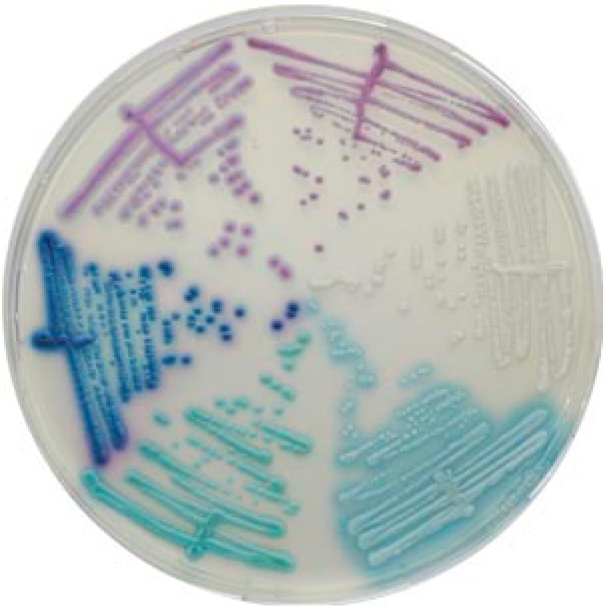

C. CHROMAgar Candida Plus (CHROMAgar France)

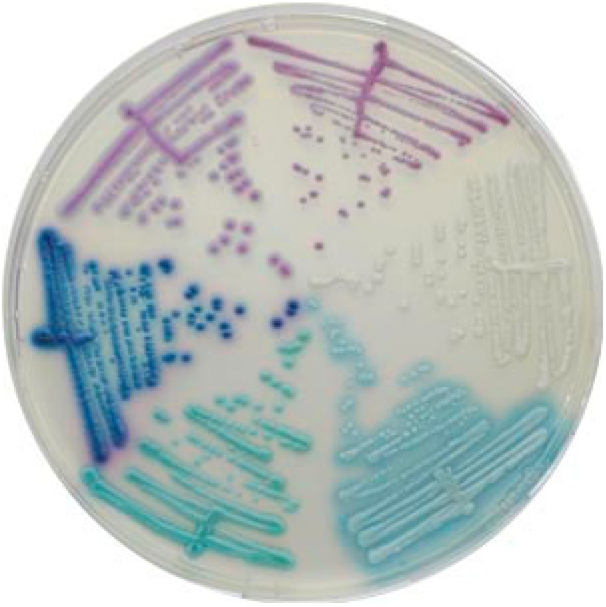

D. CHROMAgar Candida Plus (Graso)

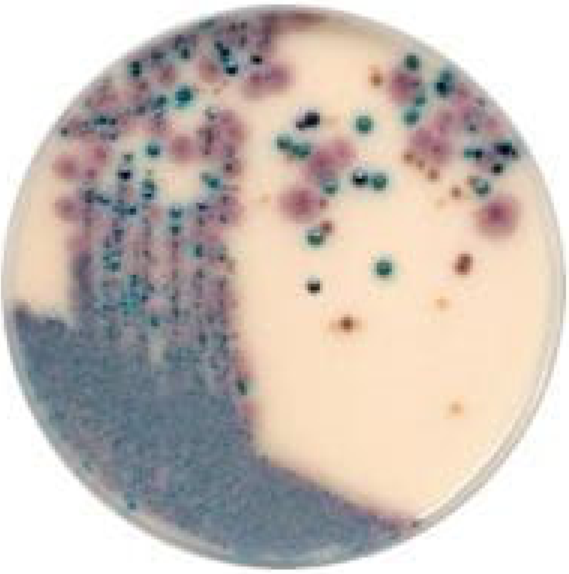

E. Brilliance Candida Agar (Oxoid)

Figure S2. Representative MALDI-TOF MS spectra for *Candida* species.

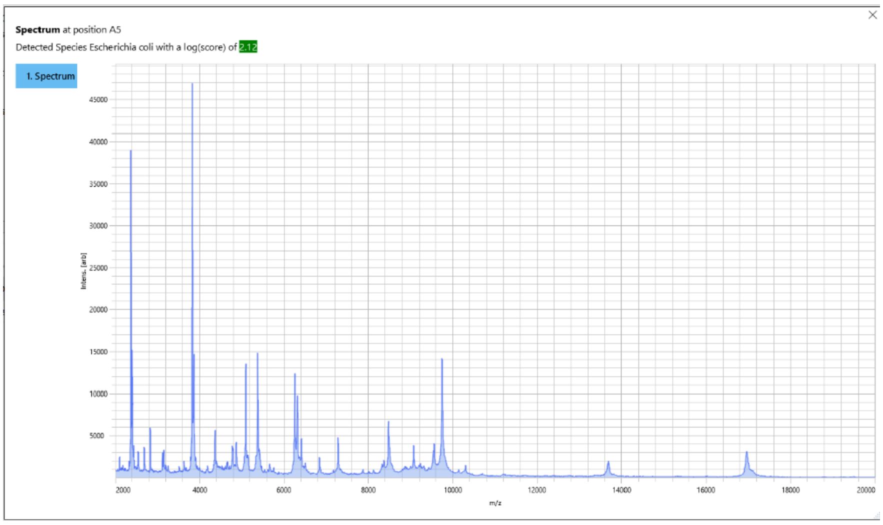

A. Bacterial Test Standard (BTS)

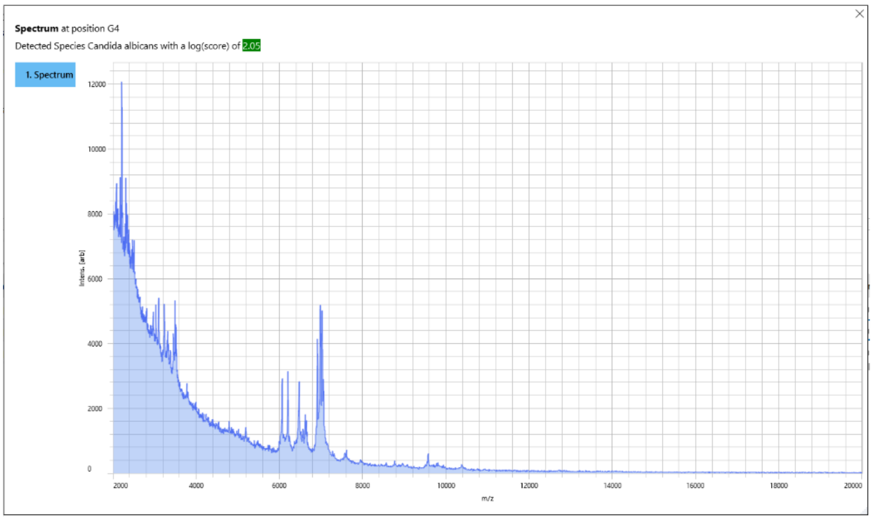

B. *Candida albicans* ATCC 90028

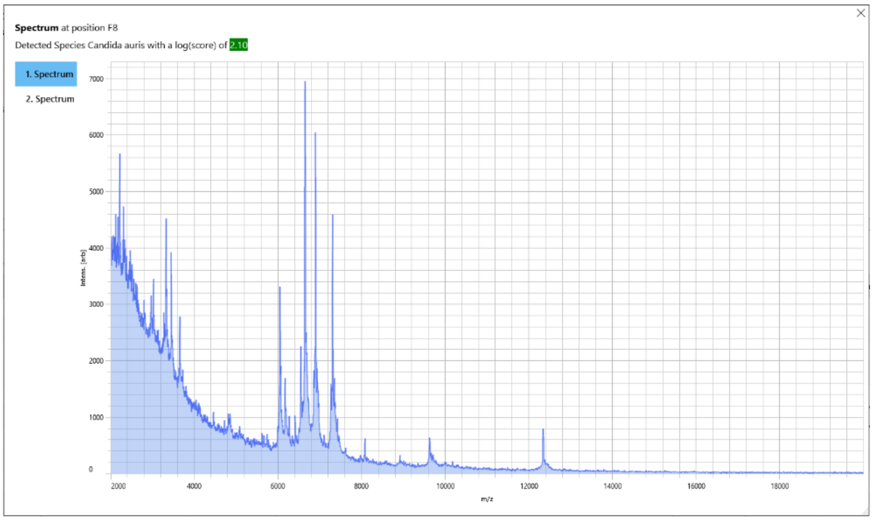

C. ***Candida auris***

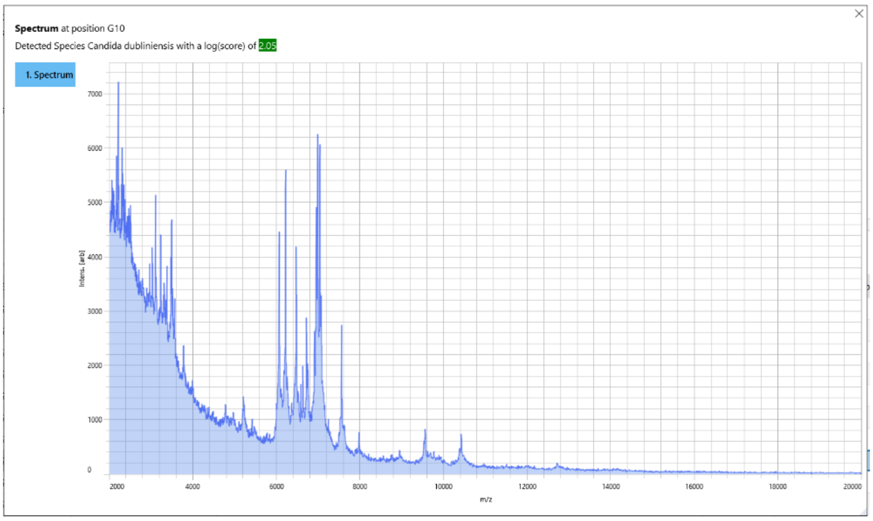

D. ***Candida dubliniensis***

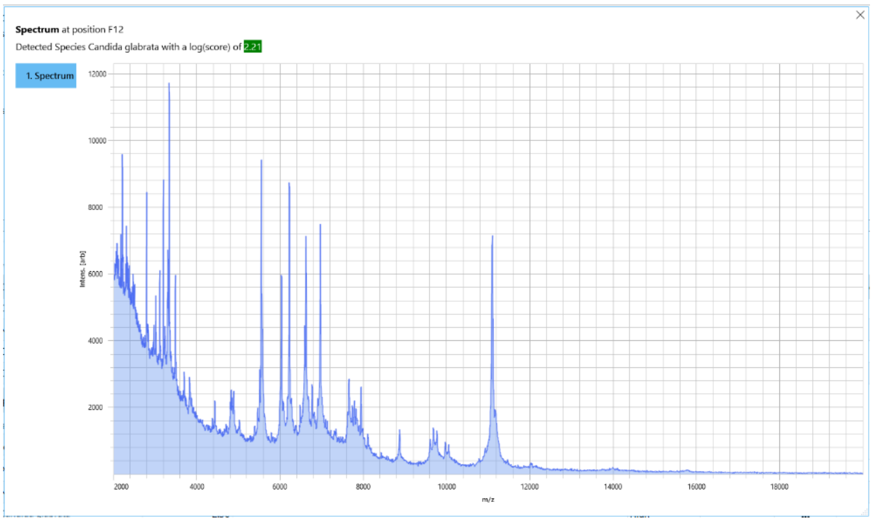

E. *Candida glabrata*

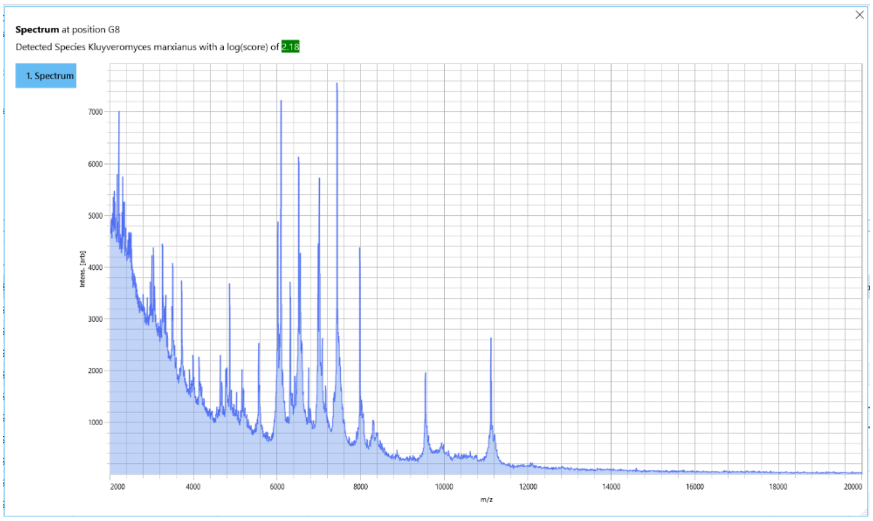

F. *Candida kefyr*

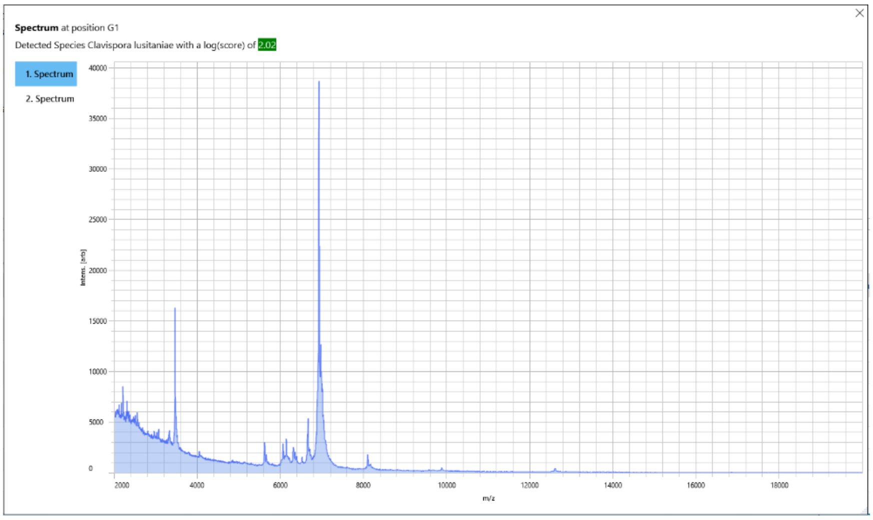

G. ***Candida lusitaniae***

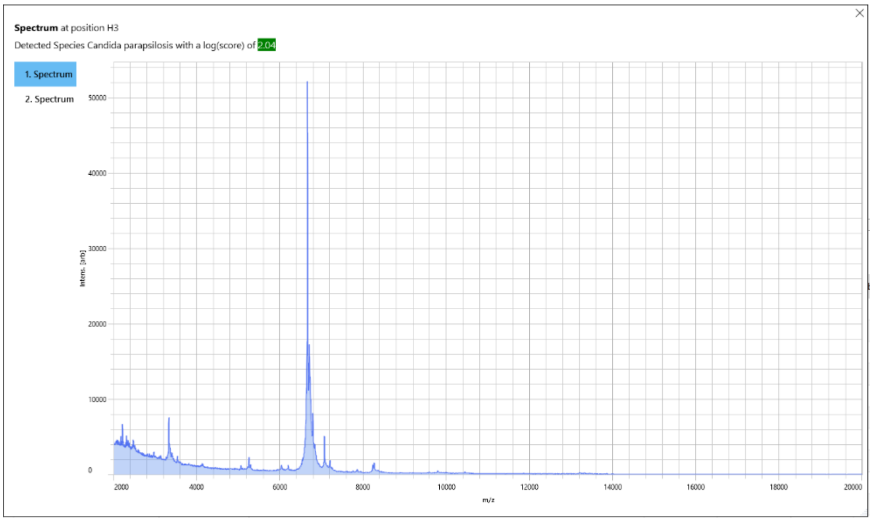

H. ***Candida parapsilosis***

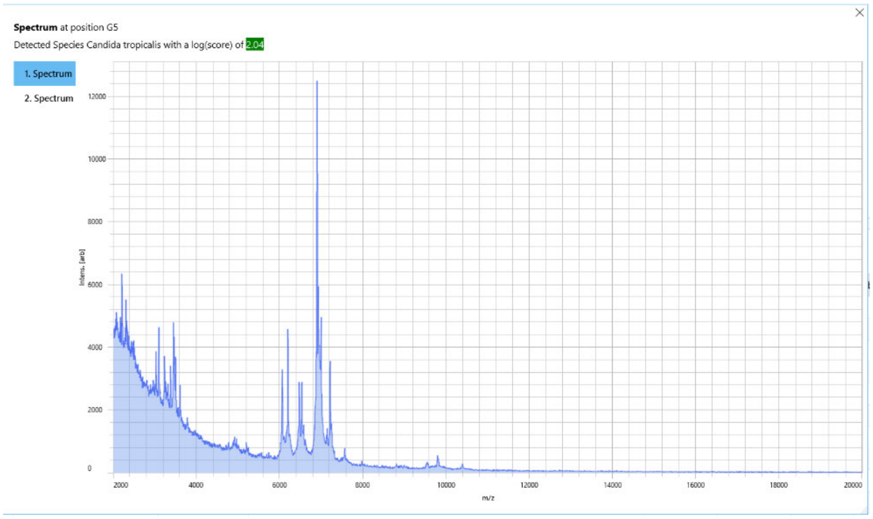

I. ***Candida tropicalis***

Figure S3. Photographs of selected cultures on BD CHROMagar Candida by Becton Dickinson; (A) *Candia albicans*, (B) *Candida dubliniensis*, (C) *Candia tropicalis*, (D) *Candida krusei.*

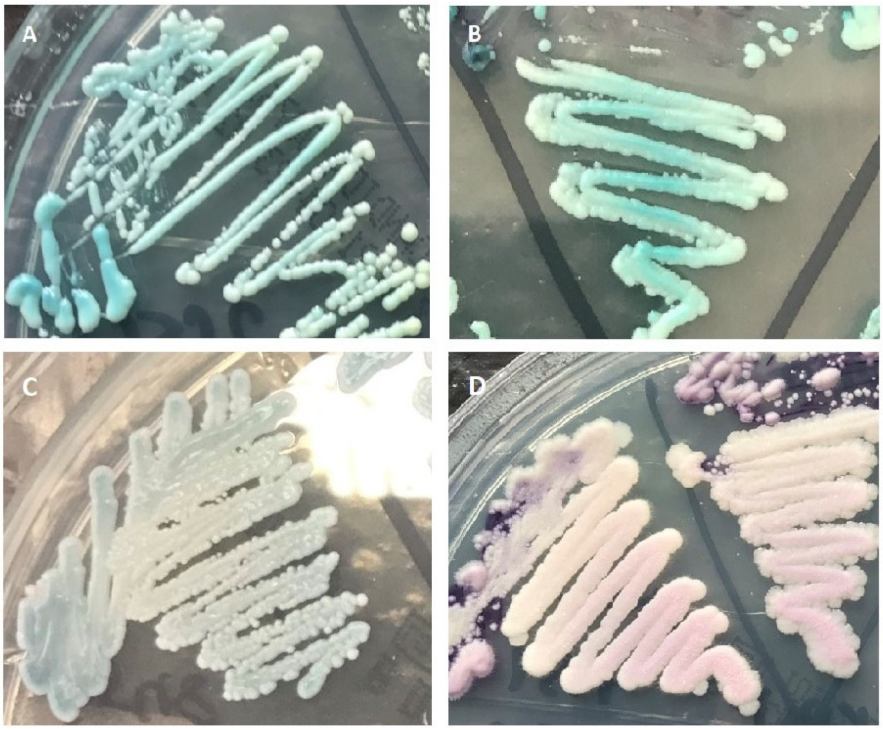

Figure S4. Photographs of chosen cultures on Agar CHROM ID Candida by *bio*Merieux; (A) *Candida albicans*, (B) *Candida dubliniensis* colonies, (C) *Candida tropicalis*, (D1) *Candida tropicalis* (D1), (D2) *Candida lusitaniae*, and (D3) *Candida kefyr*.

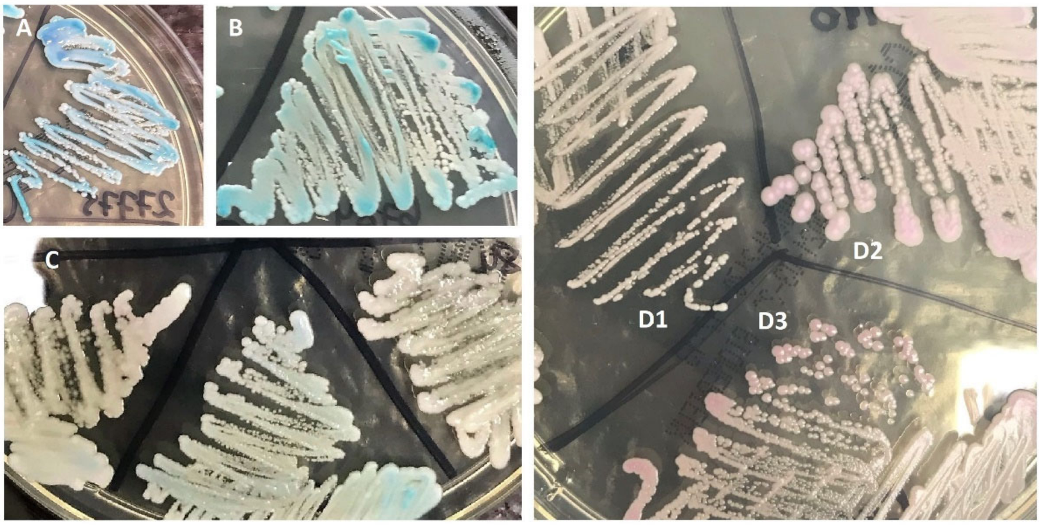

Figure S5. Photographs of chosen cultures on CHROMAgar Candida Plus by CHROMAgar France; (A) *Candida albicans*, (B) *Candida krusei*, (C) *Candida krusei*, (D) *Candida tropicalis.*

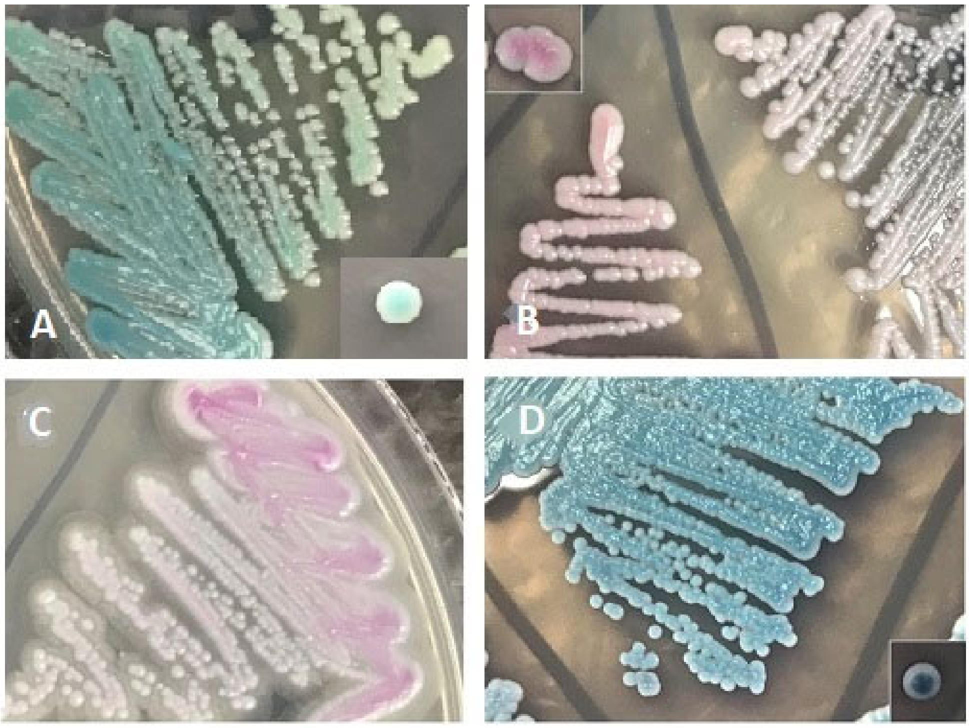

Figure S6. Photographs of selected cultures on CHROMAgar Candida Plus by GRASO Biotech; (A, B) *Candida auris*, (C, D) *Candida parapsilosis*.

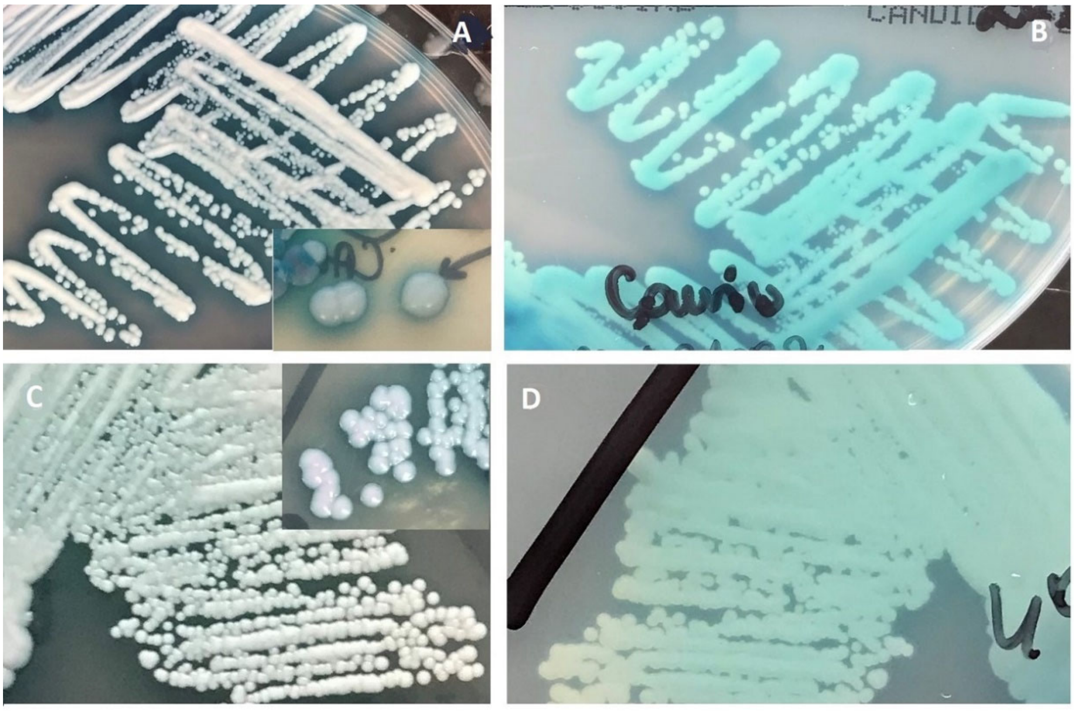

Figure S7. Photographs of some cultures on Brilliance Candida Agar by OXOID; (A) *Candida parapsilosis*, (B) *Candida kefyr*, (C) *Candida lusitaniae*, (D) *Candida krusei.*

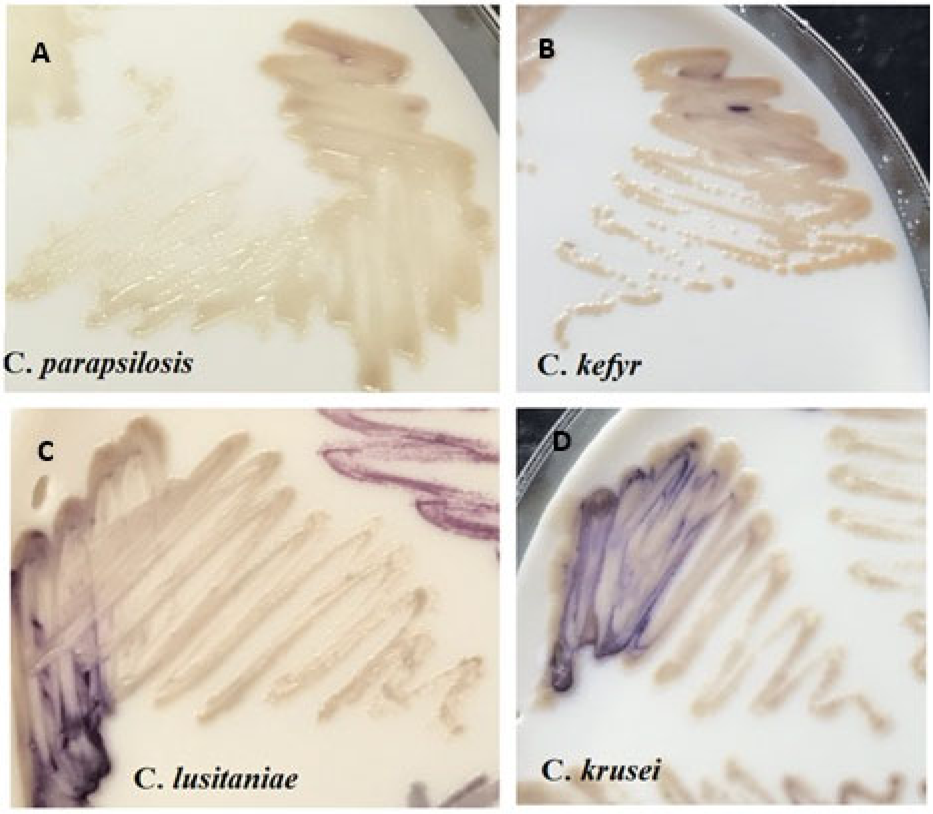

Supplement: Supplementary file 1 [file mps-08-00098-s001.zip › mps-3760737-supplementary.pdf]
